# Individual and Household Level Risk Factors Associated with Malaria in Nchelenge District, a Region with Perennial Transmission: A Serial Cross-Sectional Study from 2012 to 2015

**DOI:** 10.1371/journal.pone.0156717

**Published:** 2016-06-09

**Authors:** Jessie Pinchoff, Mike Chaponda, Timothy M. Shields, James Sichivula, Mbanga Muleba, Modest Mulenga, Tamaki Kobayashi, Frank C. Curriero, William J. Moss, for the Southern Africa International Centers of Excellence for Malaria Research

**Affiliations:** 1 Department of Epidemiology, Johns Hopkins Bloomberg School of Public Health, Baltimore, Maryland, United States of America; 2 Tropical Disease Research Center, Ndola, Zambia; Institut Pasteur, FRANCE

## Abstract

**Background:**

The scale-up of malaria control interventions has resulted in substantial declines in transmission in some but not all regions of sub-Saharan Africa. Understanding factors associated with persistent malaria transmission despite control efforts may guide targeted interventions to high-risk areas and populations.

**Methods:**

Household malaria surveys were conducted in Nchelenge District, Luapula Province, in northern Zambia. Structures that appeared to be households were enumerated from a high-resolution satellite image and randomly sampled for enrollment. Households were enrolled into cross-sectional (single visit) or longitudinal (visits every other month) cohorts but analyses were restricted to cross-sectional visits and the first visit to longitudinal households. During study visits, a questionnaire was administered to adults and caretakers of children and a blood sample was collected for a malaria rapid diagnostic test (RDT) from all household residents. Characteristics associated with RDT positivity were analyzed using multi-level models.

**Results:**

A total of 2,486 individuals residing within 742 households were enrolled between April 2012 and July 2015. Over this period, 51% of participants were RDT positive. Forty-three percent of all RDT positive individuals were between the ages of 5 and 17 years although this age group comprised only 30% of study participants. In a multivariable model, the odds being RDT positive were highest in 5–17 year olds and did not vary by season. Children 5–17 years of age had 8.83 higher odds of being RDT positive compared with those >18 years of age (95% CI: 6.13, 12.71); there was an interaction between age and report of symptoms, with an almost 50% increased odds of report of symptoms with decreasing age category (OR = 1.49; 95% CI 1.11, 2.00).

**Conclusions:**

Children and adolescents between the ages of 5 and 17 were at the highest risk of malaria infection throughout the year. School-based programs may be effective at targeting this high-risk group.

## Introduction

During the past two decades, malaria control has been a focus of the international public health community. In 2007, malaria eradication was endorsed by the Bill & Melinda Gates Foundation and later supported by the World Health Organization (WHO) and Roll Back Malaria (RBM) Partnership as a worldwide goal [1,2]. Zambia is a malaria-endemic country that rapidly scaled-up malaria control activities from 2006 to the present [3]. Unfortunately, reductions in malaria burden following scale-up of similar malaria control interventions are sometimes followed by resurgence of malaria, with a significant increase following a period of control, including in Zambia [4–6].

Malaria transmission varies across climatic seasons, ecological zones, neighboring villages, and even between neighboring households [7–10]. Households in close proximity to breeding sites have higher mosquito densities and are at increased risk of transmission, usually following a seasonal pattern [7]. However, irrigation, roads and urbanization may create breeding sites that persist throughout the year, diminishing the seasonal effect [11–14]. These environmental risk factors interact with socio-cultural factors at the level of the household, including socioeconomic status, bed net use, and the type of construction of human dwellings [10,11]. These findings suggest that despite high perennial transmission, there may be important seasonal differences in risk factors.

Despite recent reductions in malaria infection, illness, severe disease and death recorded across Zambia [15–17], malaria transmission remains high in some geographic areas. Nchelenge District is located within Luapula Province where, despite coverage with IRS with pirimiphos-methyl in 2014 (estimated 20.8% of structures sprayed in previous 12 months) and the continued distribution of LLINs (82.4% of households report having at least one LLIN) [18], the parasite prevalence by microscopy increased from 32% in 2012 to 38% in 2015 [4]. The Zambian 2011–2015 National Malaria Strategic Plan recommended targeting limited resources to the highest risk areas based on epidemiological trends [19].

Understanding individual, household and environmental factors associated with increased malaria risk is necessary to inform and optimize control strategies. Multi-level statistical techniques and spatial analysis are increasingly used to describe and assess risk factors for malaria [11,20–22]. This study analyzed a series of cross-sectional household surveys in Nchelenge District between 2012 and 2015 to identify characteristics associated with RDT positivity. Targeting control activities and distribution of preventive measures to groups or areas identified as high-risk may reduce malaria transmission in this region.

## Materials and Methods

Nchelenge District is located in northern Zambia in the marshlands of Luapula Province along Lake Mweru, and shares an international border with the Democratic Republic of the Congo. Luapula Province has the highest prevalence of malaria in children younger than five years in Zambia in 2015, 38% by microscopy and 56% by malaria rapid diagnostic test (RDT) [18]. Nchelenge District has a tropical climate with a distinct dry season (May-October) and rainy season (November-April). The population is mobile, traveling between the lake for the fishing season and inland for farming, as a fishing ban is in effect from December 1^st^ to March 1^st^.

The study procedures have been described elsewhere [23]. Briefly, a QuickbirdTM satellite image obtained from Digital Globe Services, Inc. (Denver, Colorado) was used to construct a sampling frame. One-kilometer square grid cells were overlaid on the image and cells were selected based on proximity to Lake Mweru, to ensure that households randomly selected for study enrollment were not only located along the lake. Since the population is most dense along the lake, oversampling in this ecosystem would likely have occurred. Within the selected grid cells, structures of appropriate size and shape were identified as potential households and were enumerated manually. Simple random sampling was used to select households within the grid cells that were eligible for surveys conducted throughout the year. Households were enrolled into either prospective longitudinal (visited every other month) or cross-sectional (visited once). This analysis was restricted to cross-sectional surveys and the first visit to longitudinal households because of the reduction in malaria risk following repeated study visits [24].

All individuals living at a selected residence were eligible to participate. Informed written consent was obtained from adults over the age of 16 years and parents or guardians of children younger than 16 years. A questionnaire was administered to collect demographic information, knowledge and beliefs regarding malaria transmission and prevention, history of recent malaria and anti-malarial treatment, care-seeking behavior, and the use of insecticide-treated nets (ITNs). A blood sample was collected by finger prick for a malaria rapid diagnostic test (RDT) (ICT Diagnostics, Cape Town, South Africa). Individuals who tested positive were offered treatment with artemether-lumefantrine (Coartem^®^). The study was approved by the Institutional Review Boards of the Tropical Diseases Research Centre in Ndola, Zambia and the Johns Hopkins Bloomberg School of Public Health.

### Environmental Characterization

Environmental covariates were generated based on proximity to each household’s coordinates and were analyzed using ArcGIS version 10.1 (ESRI ArcGIS, Redlands, CA). A handheld Android tablet was used to record each household’s coordinates. A digital elevation model (DEM) for the area with 90 m resolution was obtained from the Shuttle Radar Topography Mission (SRTM) version 3. The DEM was processed in ERDAS Imagine 2011. The ArcHydro Tools module of ArcGIS was used to develop a stream network and classification. This module uses elevation values to determine water flow direction and accumulation to build a stream network, which is assigned to a stream order based on the Strahler classification. The Strahler classification assigns an order value of 1, 2, 3, etc. based upon the hierarchy of tributaries. Each beginning segment of a stream or river within a stream network is a first-order or category 1, and where two first-order streams come together they form a second-order stream. When two second-order or category 2 streams meet they form a third-order stream. The degree of slope was also derived from the SRTM image.

All structures that appeared to be households in the study area were enumerated based on satellite imagery. The sum of these structures located within 500 meters of a study household was calculated as a measure of population density. The Euclidean distance from each enrolled household to the nearest road, nearest health facility and Lake Mweru were also calculated. A binary variable denoting households near the lake or interior was generated based on spatial location, with households less than 3 km from the lake considered near. The distance from Lake Mweru to the most distant site within the study area was 17 km.

The Normalized Difference Vegetation Index (NDVI) was calculated using LandSat 5 data and is calculated as: (near infrared—visible infrared) divided by (near infrared+visible infrared) (μm). The values correlate with density of vegetation. NDVI was explored as a continuous variable but a binary NDVI variable was used for the statistical models due to lack of variation in the study area. Rainfall data from a HOBO Micro Station (Onset Computer Corporation, Bourne, MA) was used to generate a binary variable for season. The HOBO is a four-sensor data logger designed to measure rainfall, temperature, and relative humidity at hourly intervals.

### Statistical Analyses

RDT positive individuals were compared to RDT negative individuals for individual level characteristics. Household level characteristics were explored comparing households with no RDT positive residents to those with any RDT positive residents. Proportions were tested using the chi-square test and means were compared using Student’s t-test. The effects of individual and household level characteristics on malaria risk were analyzed using multi-level logistic regression models. All Euclidean distance variables were multiplied by -1 to show increased risk in relation to closer proximity to the ecological variable. Multi-level models with regression inference based on generalized estimating equations (GEE) were used to account for the data hierarchy and clustering of individuals nested within households. For analysis, environmental variables were considered at the same data level as households but labeled separately for purposes of interpretation. Two-way interaction terms were generated to assess seasonal variation in the effects of each environmental risk factor. Variables with p values ≤0.1 in univariate models were eligible for inclusion in the final multivariate model and only retained if at the stricter 0.05 significance level. Statistical analyses were carried out using SAS software version 9.3 (SAS Institute, Cary, NC).

## Results

**Fig 1:**
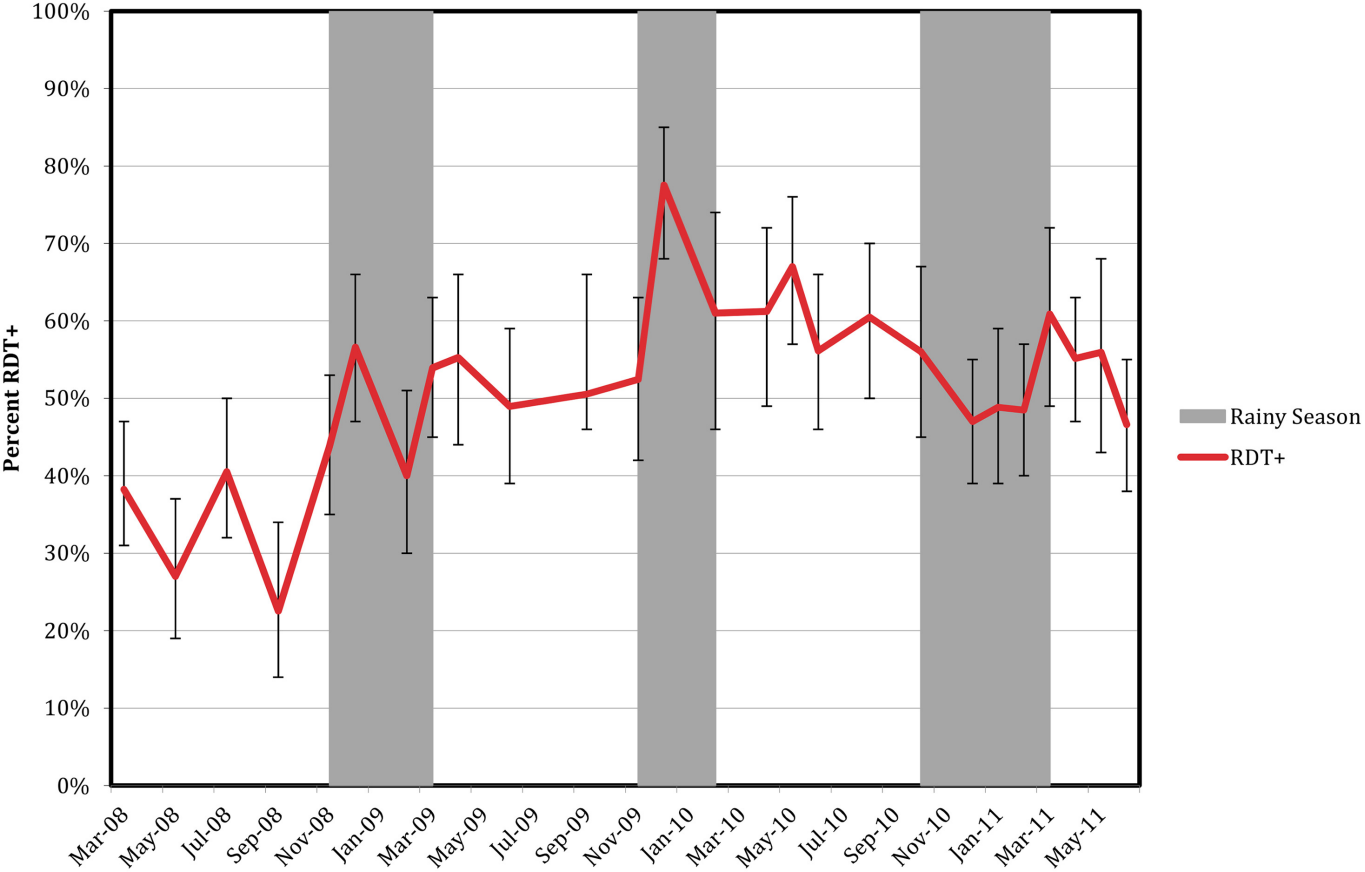
Percent of enrolled participants who were RDT positive per month in Nchelenge District, Zambia from April 2012 to July 2015.

**Fig 2:**
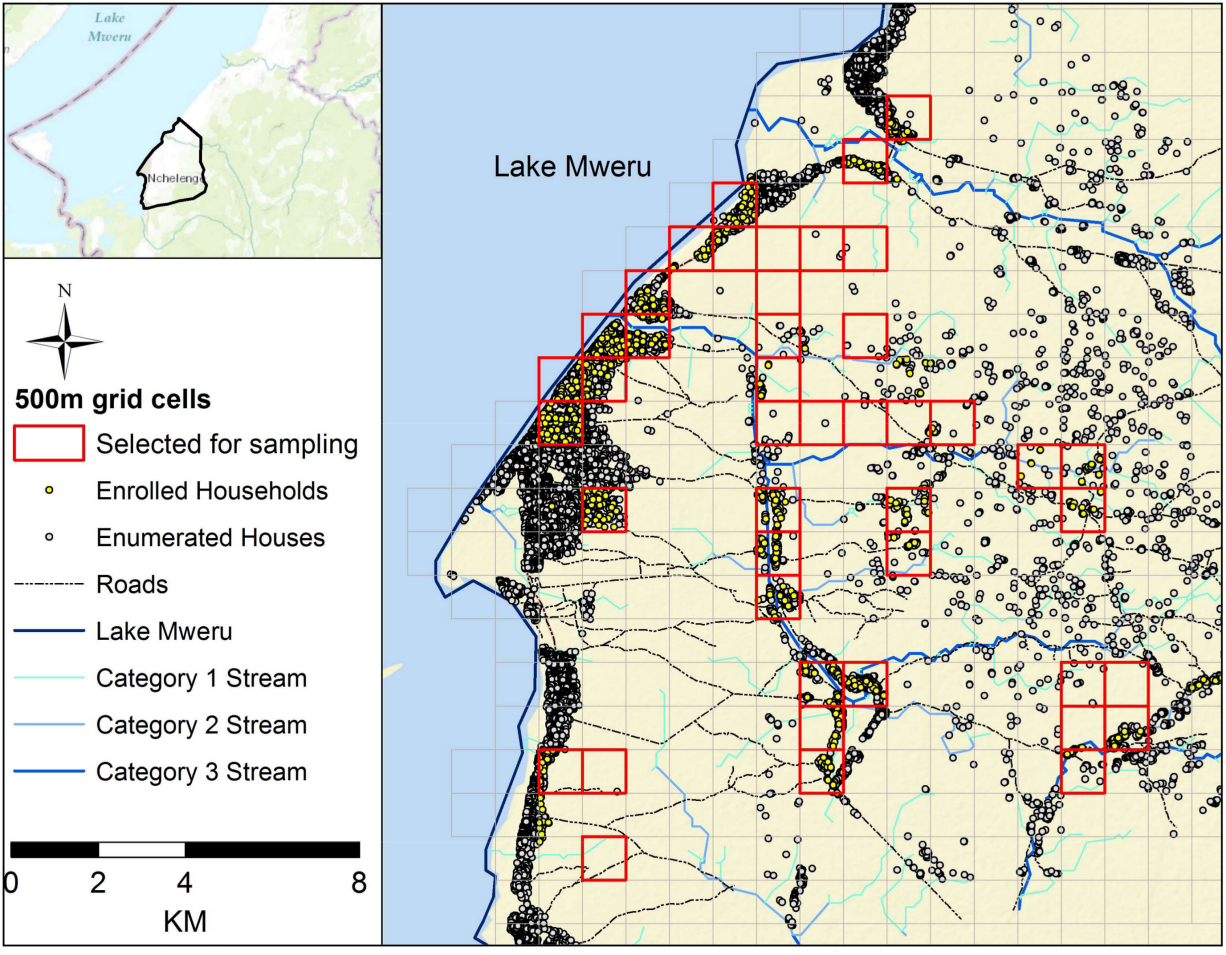
Map of 1 km grid cells, enumerated structures and enrolled households in Nchelenge District, 2012–2015.

A total of 2,486 individuals residing in 742 households were visited between April 2012 and July 2015. Parasite prevalence as determined by RDT was 38% in 2012 and increased to 52% in 2015 (Fig 1). Despite seasonal rainfall, RDT positivity remained higher than 20% every month (Fig 1). The locations of enumerated households, enrolled households, and the HOBO monitoring station are depicted (Fig 2).

Several individual level factors were associated with RDT positivity in univariate analyses. A higher proportion of RDT positive individuals were 5 to 17 years old (43%) compared to children younger than 5 years (24%) and adults older than 18 years (28%) (Table 1). RDT positivity was associated with reported symptoms within the prior 2 weeks (48% vs 41%, p<0.0001) (Table 1). Reported ownership of an ITN the night before was lower for RDT positive individuals (78%) compared to RDT negative individuals (85%), consistent with a modest protective effect (p<0.0001) (Table 1).

**Table 1:** Individual characteristics associated with RDT positivity in Nchelenge District, 2012–2015.

	RDT positive	RDT negative	Univariate unadjusted p-value
Number of individuals	1,256	1,230	
Female	658 (52%)	710 (58%)	0.008
Age category			<0.0001
< 5 years	295 (24%)	196 (15%)	
5 to 17 years	542 (43%)	206 (17%)	
≥18 years	347 (28%)	793 (65%)	
Missing	72 (6%)	35 (3%)	
Last time visited health facility for malaria			0.01
1 months ago	288 (23%)	260 (21%)	
2–6 months ago	398 (32%)	374 (30%)	
≥ 7 months ago	252 (20%)	319 (26%)	
Never	200 (16%)	181 (15%)	
Missing	118 (9%)	96 (8%)	
Experienced symptoms in past month (fever with headache or chills)	606 (48%)	510 (41%)	<0.0001
Took antimalarials in past month	292 (23%)	223 (18%)	0.01
Missing	2 (0%)	5 (0%)	
Own ITN	979 (78%)	1,045 (85%)	<0.0001
Use ITN (if owned)	646 (51%)	823 (67%)	<0.0001

Several household level factors were associated with having at least one RDT positive resident compared to households with no RDT positive residents. As expected, households comprised of a higher number of residents were more likely to have at least one RDT positive resident (median 4 vs. 2 household members), reflecting the higher number of individuals in the household at risk (Table 2). Reported use of an open well as the main source for household drinking water was higher among households with any RDT positive residents (37% vs. 28%). Households in areas with lower population density and in the interior away from Lake Mweru were more likely to have an RDT positive resident (median 170 structures within 500 m of the household vs. 278 structures) (Table 2).

**Table 2:** Household characteristics with and without RDT positive residents in Nchelenge District, 2012–2015.

	At least 1 RDT+ household member	No RDT+ household members	Univariate unadjusted P Value
	N (%)		
Households	535	207	
Median number household members (lower, upper)	4 (2, 5)	2 (1, 2)	<0.0001
Ever received IRS	177 (33%)	74 (36%)	0.46
Missing	10 (2%)	5 (2%)	
Open well	198 (37%)	57 (28%)	0.02
Median elevation in meters (IQR)	959 (948, 968)	959 (950, 968)	0.49
Median distance in meters to category 1 stream (IQR)	533 (235, 920)	590 (265, 900)	0.003
Median distance in meters to category 2 stream (IQR)	1719 (576, 2644)	2012 (613, 2656)	0.02
Median distance meters to category 3 stream (IQR)	1123 (301, 2097)	1648 (406, 2265)	0.13
Median distance in meters to road (IQR)	73 (38, 135)	81 (43, 154)	0.07
Degree of slope (IQR)	1.7 (1.1, 2.5)	1.6 (1.0, 2.0)	0.48
Percent inland (3 km from Lake Mweru)	326 (61%)	146 (71%)	0.01
NDVI* value ≥ 0.5	279 (52%)	89 (43%)	0.04
Number of other households within 500 m of each household	170 (78, 422)	278 (103, 579)	<0.0001
Study visit in rainy season	262 (49%)	1014 (50%)	0.85

*NDVI = normalized difference vegetation index

In a multi-variable GEE analysis, several individual and household characteristics were associated with RDT positivity. Compared to adults over the age of 18 years, children younger than five years had 3.39 higher odds (OR = 3.39; CI 2.24, 5.13) of being RDT positive, and children 5 to 17 years of age had almost nine times higher odds (OR = 8.83; CI 6.12, 12.71) of being RDT positive (Table 3). As expected, history of fever was significantly associated with RDT positivity and approached significance in a two-way interaction with age: as the age category decreased a history of fever increased 49% (OR = 1.49; CI 1.11, 2.00). Other individual level variables, such as receiving malaria medications in the past 2 weeks or using an ITN, that were significant in the univariate analyses, were not significantly associated with RDT positivity in the full GEE model.

**Table 3:** Factors associated with RDT positivity in Nchelenge District, 2012–2015.

Factors	Univariate	Multivariable
	Odds Ratio (95% CI)	Odds Ratio (95% CI)
**Fixed Effects**		
**Individual Level Factors**		
Female	**0.81 (0.69, 0.95)**	0.88 (0.69, 1.11)
Age Category		
0 to 4 years	**3.44 (2.76, 4.28)**	**3.39 (2.24, 5.13)**
5 to 17 years	**6.01 (4.90, 7.36)**	**8.83 (6.13, 12.71)**
Older than 18 years	REFERENCE	REFERENCE
Symptoms (fever with headache or chills)	**1.31 (1.12, 1.54)**	0.85 (0.47, 1.53)
Age symptoms interaction°	**1.24 (1.00, 1.55)**	**1.49 (1.11, 2.00)**
Sought care for malaria past 6 months	**0.90 (0.83, 0.97)**	0.95 (0.84, 1.08)
Taken antimalarials past 4 weeks	**1.37 (1.13, 1.67)**	0.80 (0.58, 1.10)
Use ITN night before	**0.38 (0.32, 0.46)**	0.83 (0.61, 1.13)
**Household and Environmental Level Factors**		
Water source: open well	1.23 (0.96, 1.56)	1.00 (0.73, 1.38)
IRS, ever	0.86 (0.67, 1.11)	1.05 (0.76, 1.44)
Elevation (per 10 m)	**0.94 (0.88, 0.99)**	**0.85 (0.77, 0.95)**
Degree of slope	1.02 (0.95, 1.10)	-
Distance to nearest road*	**0.34 (0.14, 0.53)**	**1.49 (1.19, 1.85)**
Within 3 km of Lake Mweru	**0.76 (0.60, 0.96)**	1.12 (0.63, 1.98)
Number of structures within 500 m (per 25 structures)	**0.97 (0.96, 0.98)**	**0.95 (0.93, 0.97)**
Rainy season	1.09 (0.93, 1.28)	1.14 (0.86, 1.51)
NDVI value ≥ 0.5	**1.36 (1.08, 1.72)**	1.09 (0.81, 1.47)
Distance to category 1 stream*	**1.10 (1.03, 1.17)**	1.08 (0.99, 1.17)
Distance to category 2 stream*	**1.03 (1.01, 1.06)**	1.03 (0.97, 1.08)
Distance to category 3 stream*	1.02 (0.99, 1.04)	0.99 (0.95, 1.03)
**Random Effects**		
Household random effect	-	0.85 (0.22)

* Per 250 meters

° Interaction term between age and symptoms. So, for each increase in age category, there is a reduced reporting of symptoms.

**Fig 3:**
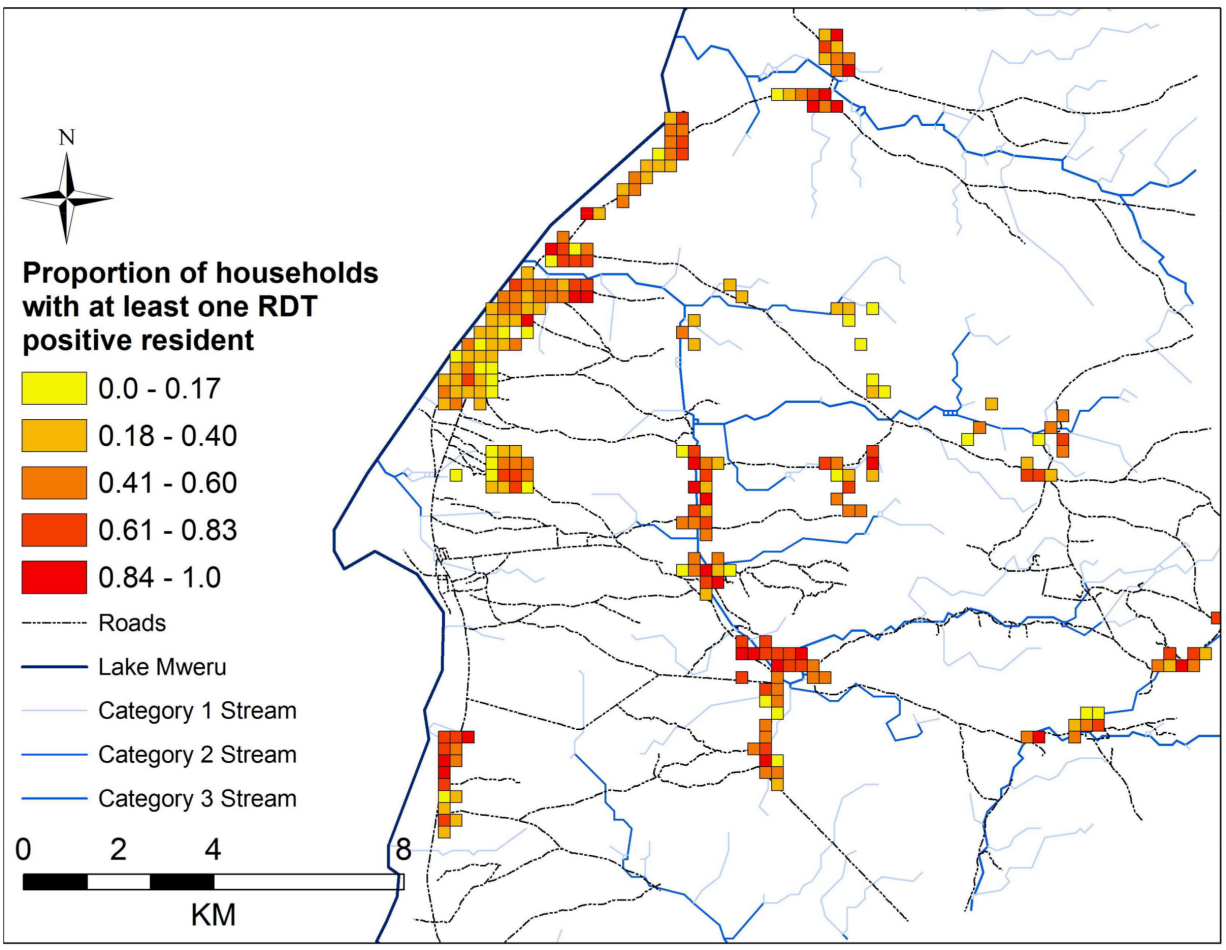
Map of study households aggregated to 250 meter grid cells showing the proportion of study households in each grid cell with at least one RDT positive resident in Nchelenge District, Zambia from 2012 to 2015.

Several household level factors were associated with RDT positivity in the multivariable GEE model. The median elevation in the study area was 951 meters (minimum 877 meters, maximum 1049 meters). As household elevation increased by 10 meters, the odds of RDT positivity decreased 15% (OR = 0.85; CI 0.77, 0.95) (Table 3). Households located closer to roads had 49% higher odds of having an RDT positive resident (OR = 1.49; CI 1.19, 1.85). Households located inland in proximity to streams in the less population dense areas had higher probability of RDT positive residents compare with those by the lake. As the area around a house increased in population density (increase per 25 additional structures), the odds of an RDT positive resident decreased (OR = 0.95; CI: 0.93, 0.97). Since the household level random intercept variance estimate was larger than the individual level random intercept variance estimate, greater differences between households were observed than the variation between individuals within a household. All study households were aggregated to 250-meter grid cells overlaid on the study area, and the proportion of study households that had at least one RDT positive resident are presented by grid cell. The map highlights that a higher proportion of households per grid cell that are RDT positive appear to be located inland, with variation along the lake (Fig 3).

## Discussion

Despite the rollout of malaria control interventions, Nchelenge District experiences intense perennial transmission of malaria. This study identified school-age children as a high-risk population and identified several household and environmental factors that may increase transmission risk. School-age children were the most likely to have malaria but also less likely than young children to experience symptoms, presumably due to the acquisition of clinical immunity [25,26]. Proximity to certain environmental features, such as roads, lower population density and lower elevation may increase household malaria risk because they are generally predictive of anopheline mosquito breeding sites [10,27]. These individual and household level risk factors may explain minor variations in malaria risk.

There are several reasons malaria control efforts may be ineffectual in this setting. The recent rise in reported cases could be attributed to increasing insecticide resistance, population movement across borders from high transmission areas, shifts in biting behavior of mosquitoes to outdoors or earlier times, or increased use of RDTs for parasitological confirmation [4,16,28]. Additionally, while delivery of health services and key interventions has increased, coverage levels may be too low to have a measurable impact [4,29]. For example, according to the 2015 National Malaria Indicator Survey (MIS), Luapula Province reported low rates of IRS (only 25.9% of households were sprayed in the previous 12 months) [18]. Importantly, LLIN use in Luapula Province increased from 34% in 2010 to 72.5% in 2015 [18,30].

Interestingly, the odds of a positive RDT were higher closer to roads. This may be because erosion along dirt roads created puddles that are ideal for breeding sites, or may be indicative of travel and increased risk of imported malaria. A study in Kenya found that water pooled along roads, creating potential breeding sites [13]. Testing for anopheline mosquito larvae along roads may identify opportunities for targeted vector control interventions. Streams in this area may also be predictive of anopheline breeding sites [31]. Although streams were significantly associated with household RDT positivity in univariate analyses here and in environmental models [23], these associations were not significant after controlling for individual level factors such as age.

In addition to environmental factors associated with RDT positivity, some individual and household level characteristics were also significant. Age of the participant was highly associated with RDT positivity, in particular among children aged 5 and 15 years. Children in this age category are at increased risk of malaria and are least likely to use interventions such as LLINs [8,32]. Priority for LLINs is often given to very young children and women of child bearing age, leaving school-aged children vulnerable. Age was also found to interact with reported fever. As age increased, reported fever decreased. Previous studies have identified an interaction between age, report of fever, lower parasitemia and season [33]. A high prevalence of asymptomatic parasitemia may be critical in maintaining transmission [34–36]. School-aged children who have acquired clinical immunity may be asymptomatic carriers of both asexual parasites and gametocytes, and may be reservoirs for the parasite through the dry season [37]. School based test and treat interventions and/or seasonal malaria chemoprophylaxis (SMC) may be particularly effective in reducing transmission in Nchelenge District.

This analysis has some limitations, mainly that it is reliant on RDTs to detect infection with *P*. *falciparum*. In such a high transmission setting, some people may be antigenemic after parasite clearance, leading to over-estimation of parasitemia by RDT [38]. According to the 2015 MIS, 38% of children younger than five years of age had malaria parasites by microscopy and 55.6% had malaria by RDT [18]. Lastly, it is likely that travel occurs in this area, for migrant work such as fishing or farming, or social functions such as weddings and funerals, which may present opportunity for existence of malaria infection not related to the participants’ household.

Despite seasonal rainfall, perennial transmission of malaria continues in Nchelenge District. In resource constrained settings, targeted control interventions and novel strategies are necessary to reduce transmission [39–41]. First, malaria risk maps may be useful in targeting vector control activities to high-risk geographic areas. Second, school-based interventions should be explored to target this high-risk population. The epidemiology and management of malaria in school-age children has received little attention but has consequences on educational attainment and transmission [26]. IPT is now being studied in school-age children in the form of intermittent parasite clearance in schools (IPC), seasonal malaria chemoprevention (SMC), and test and treat programs administered by schools [26]. Targeting high-risk geographic areas and populations may be cost effective and efficient for reducing malaria transmission.
